# A systematic search for SNPs/haplotypes associated with disease phenotypes using a haplotype-based stepwise procedure

**DOI:** 10.1186/1471-2156-9-90

**Published:** 2008-12-22

**Authors:** Yin Yang, Shuying Sue Li, Jason W Chien, Jessica Andriesen, Lue Ping Zhao

**Affiliations:** 1 Division of Public Health Sciences, Fred Hutchinson Cancer Research Center, 1100 Fairview Ave. N, Seattle, WA 98109, USA; 2 Division of Clinical Research Sciences, Fred Hutchinson Cancer Research Center, 1100 Fairview Ave. N, Seattle, WA 98109, USA; 3 Fred Hutchinson Cancer Research Center, 1100 Fairview Ave. N, Mailstop M2-B500, Seattle, WA 98109, USA

## Abstract

**Background:**

Genotyping technologies enable us to genotype multiple Single Nucleotide Polymorphisms (SNPs) within selected genes/regions, providing data for haplotype association analysis. While haplotype-based association analysis is powerful for detecting untyped causal alleles in linkage-disequilibrium (LD) with neighboring SNPs/haplotypes, the inclusion of extraneous SNPs could reduce its power by increasing the number of haplotypes with each additional SNP.

**Methods:**

Here, we propose a haplotype-based stepwise procedure (HBSP) to eliminate extraneous SNPs. To evaluate its properties, we applied HBSP to both simulated and real data, generated from a study of genetic associations of the bactericidal/permeability-increasing (BPI) gene with pulmonary function in a cohort of patients following bone marrow transplantation.

**Results:**

Under the null hypothesis, use of the HBSP gave results that retained the desired false positive error rates when multiple comparisons were considered. Under various alternative hypotheses, HBSP had adequate power to detect modest genetic associations in case-control studies with 500, 1,000 or 2,000 subjects. In the current application, HBSP led to the identification of two specific SNPs with a positive validation.

**Conclusion:**

These results demonstrate that HBSP retains the essence of haplotype-based association analysis while improving analytic power by excluding extraneous SNPs. Minimizing the number of SNPs also enables simpler interpretation and more cost-effective applications.

## Background

Genotyping technology now enables population researchers to genotype dozens to thousands of SNPs within any selected candidate gene or within any genomic region. Such SNP data are increasingly collected in disease association studies, using a case-control study design [1,2], with the analytic objective of assessing association between SNP genotypes and a disease phenotype of interest. While traditional analyses have involved correlating phenotypes with individual SNP genotypes [3], a complementary approach involves inferring haplotypes of SNPs or their distributions, then assessment of haplotypic associations with the disease phenotype [4-7].

Haplotype-based association analysis has several advantages over association analysis with single SNPs. First, haplotypes of multiple SNPs can reduce the number of comparisons to be made during the analysis. Typically, SNPs within a narrow region are in high LD, i.e., adjacent SNP alleles on the same chromosome are highly correlated. Consequently, with *K *such SNPs, the total number of haplotypes formed by these SNPs is generally much smaller than the number of all possible haplotypes (= *2*^*K*-1^). For a typical gene, the number of common haplotypes, even with variable numbers of SNPs, is on the order of 10–15 [8], with a few notable exceptions such as the major histocompatibility (MHC) genes [9]. Secondly, a haplotype is naturally interpreted as genetic polymorphisms of SNP alleles on the same chromosome. After filling in the non-SNP nucleotides between SNPs, one has a fully phased DNA sequence. This sequence, if it lies in the coding region of a gene, can be converted into an amino acid sequence, and thus haplotypic variations may result in protein variations, an important biological context to consider. Third, haplotypes themselves tend to be conserved and shaped by evolutionary processes. Recent population genetic studies of the human genome have suggested that recombination processes, together with other population genetic forces, have created long-range haplotype blocks [10,11]. These block structures are also useful for reducing the number of statistical comparisons, as well as for interpretation of disease associations with common extended haplotypes. Additionally, haplotype-based associations are useful for mapping unknown disease mutations. As opposed to assuming a direct relationship between a phenotype and an individual SNP, one or more disease-causing mutations may be in high LD with adjacent SNPs; hence, extended haplotypes formed by these known SNPs may serve as markers for disease-causing mutations yet to be discovered [12]. Indeed, a haplotype of multiple SNPs may be thought of as an allele at a multi-allelic marker locus, and increasing polymorphism with multiple haplotypes improves the power to detect disease associations.

There are also disadvantages when using haplotype-based association analyses: the main disadvantage is that haplotype-based association analyses may have reduced power in detecting SNP-level associations. If in truth, the disease association is with a single functional SNP, the haplotype-based association can be less efficient due to the fact that including irrelevant SNPs effectively divides the samples into multiple haplotype groups, hence reducing sample sizes and consequently decreasing the power of the study. Moreover, dividing a single SNP association into multiple haplotype associations will also incur the penalty associated with multiple comparisons. This loss of statistical efficiency is exacerbated if the haplotype analysis includes many SNPs, resulting in an excessively large number of haplotypes.

To retain the advantages of haplotype-based analysis and overcome its disadvantages, we propose a HBSP to systematically search for a subset of SNPs whose haplotypes associate with the disease phenotype. Following the principle of stepwise regression methodology, for example, forward or backward selection [13], we have developed forward and backward haplotype-based stepwise procedures. For example, in the backward procedure, one gradually de-selects one SNP at a time, provided that the exclusion of individual SNPs does not alter the observed haplotypic association. In this report, we introduce the methodology of the HBSP, report our results from simulation studies with finite sample sizes, and illustrate its clinical applicability by using the HBSP to select functional SNPs within the BPI gene, which has been independently shown to be significantly associated with pulmonary function in post-transplant patients.

## Methods

### BPI and Pulmonary Function among Transplant Patients

Given the importance of innate immunity in protection from diseases of the airway, we conducted a genetic association study using a candidate gene approach to determine if polymorphisms in genes of the innate immune pathway are associated with the development of hematopoietic stem cell transplant- (HCT-) related airflow obstruction (AFO), the details of which have been published elsewhere [14]. This two-tiered (including discovery and validation phases) case-control genetic association study selected tagging SNPs from 15 genes from the innate immunity pathway. Cases were defined as patients who experienced an annual decrease of the forced expiratory volume in the first second (FEV_1_) > 5%, with their lowest post-transplant ratio of FEV_1 _to forced vital capacity < 0.8. This study discovered and validated the association of multiple tagging SNP haplotypes on the BPI gene with the AFO phenotype. In the analyses below, eight tagging SNPs from the BPI gene are used for illustrative purposes.

### Notation, Model and Estimation Procedures

#### Notation

Consider a case-control study with *n *subjects (*i *= 1,2,..., *n*), with cases denoted by *d*_*i*_*= 1 *and controls denoted as *d*_*i*_*= 0*. Let *x*_*i *_= (*x*_*i*1_, ...*x*_*ic*_)' denote a vector of *c *covariates, such as clinical, demographical, and lifestyle variables. Also obtained from the *i*th subject is a biological sample, which is genotyped for multiple SNPs. Let *g*_*i *_= (*g*_*i*1_, *g*_*i*2_, ...,*g*_*iq*_) denote genotypes of linearly ordered SNPs within a well-defined genomic region, such as a candidate gene region. Let *g*_*ij *_= *a*_*ij*_: ${\dot{a}}_{ij}$ denote a pair of alleles at the *j*th locus in the *i*th individual, where bi-allelic SNP alleles *a*_*ij *_and ${\dot{a}}_{ij}$ take a value of 0 and 1, corresponding to the major and minor allele, respectively. Due to the nature of the genotyping technology, the parental origin (or phase) of individual alleles is unknown. Let Ω_*i *_= (Ω_*i*1_, Ω_*i*2_, ..., Ω_*iq*_) denote a vector of phase indicators: Ω_*ij *_= 0 implies that the first allele at the *j*th locus for the *i*th subject *a*_*ij *_is inherited from the father, with the other allele, ${\dot{a}}_{ij}$, from the mother. In contrast, Ω_*ij *_= 1 implies that *a*_*ij *_is inherited from the mother and ${\dot{a}}_{ij}$ from the father. When phases are known, (*g*_*i*_, Ω_*i*_) defines two haplotypes called a diplotype, denoted as *H*_*i *_: ${\dot{H}}_{i}$. Each haplotype consists of *q *SNPs and may be written as *H*_*i *_= *a*_*i*1_*a*_*i*2_*a*_*i*3_...*a*_*iq*-1_*a*_*iq*_. For *q *SNPs, there are *r *possible haplotypes, denoted as (*h*_1_, *h*_2_, ..., *h*_*r*_).

The penetrance of haplotypes and covariates to the disease phenotype is quantified through a logistic regression model. The logistic penetrance function can be formally written as

(1) Pr⁡(di=1|Hi,H˙i,xi)=11+exp⁡{−α−β˜'[K(Hi)+K(H˙i)]−γ˜'xi},

which takes values between 0 and 1, quantifying the probability of being diseased. The *K*(·) is a vector of (*r*-1) indicator functions, i.e *K*(*H*_*i*_) = (*I*(*H*_*i *_= *h*_2_), *I*(*H*_*i *_= *h*_3_), ..., *I*(*H*_*i *_= *h*_*r*_))' where the haplotype *h*_1 _is treated as a reference. The unknown regression coefficient vector  = (β1, β2,..., βr-1)' quantifies haplotype-specific penetrance to the phenotype and is estimated from $\underset{˜}{\beta }$ the data, along with other unknown regression parameters, the coefficient of intercept *α *and the coefficients of the other covariates $\underset{˜}{\gamma }$. The regression coefficient *β*_*j *_is also referred as the logarithm of odds ratio (OR). The estimation algorithm for parameters and their covariates were developed elsewhere [4].

#### A Wald Test Statistic

In our proposed HBSP described below, we either add (in forward selection) or remove (in backward selection) one SNP at each time. Suppose that there are *q *SNP loci with *r *different haplotypes denoted as (*h*_1_, *h*_2_, ..., *h*_*r*_) considered in the above logistic regression model. The estimations of $\underset{˜}{\beta }$ and its covariance $\Sigma_{\underset{˜}{\beta }}$ are denoted by $\underset{˜}{\hat{\beta }}=({\hat{\beta }}_{1},{\hat{\beta }}_{2},{\hat{\beta }}_{3},...,{\hat{\beta }}_{r-1})$ and ${\hat{\Sigma }}_{\underset{˜}{\beta }}$.

To examine the contribution of a particular SNP to the overall haplotypic association, we remove one SNP at a time from the haplotypes. For example, if the *q*th SNP is removed, some of the haplotypes may be merged if their haplotypic differences were due to allelic difference at the *q*th SNP. To assess the contribution of the *q*th SNP to the disease association, it is equivalent to test if the coefficients of merged haplotypes are equal. Suppose *s *unique haplotypes are observed after removing the *q*th SNP. So (*r-s*) haplotypes are merged with one of *s *unique haplotypes, thus number of equalities to be tested is (*r-s*).

Under the null hypothesis that the *q*th SNP has no contribution to the haplotype-based association, we thus compute haplotype-based parameters for *s *haplotypes with (q-1). Further, under the null hypothesis, one could use estimated *s *haplotype-based parameters to assign haplotype-based parameters for all *r *haplotypes, as if *q *SNPs been included. Let $\tilde{\underset{˜}{\beta }}=({\tilde{\beta }}_{1},{\tilde{\beta }}_{2},...,{\tilde{\beta }}_{r-1})$ denote such haplotype-based parameters obtained under the null hypothesis with the *q*th SNP removed.

To test whether the *q*th SNP contributes significantly to the haplotype-based association, we construct the following Wald statistic:

(2) $$\chi_{r-s}^{2}=(\underset{˜}{\hat{\beta }}-\underset{˜}{\tilde{\beta }})'\Sigma_{\underset{˜}{\tilde{\beta }}}^{-1}(\underset{˜}{\hat{\beta }}-\underset{˜}{\tilde{\beta }}),$$

where $\Sigma_{\underset{˜}{\tilde{\beta }}}$ is the estimated covariance matrix of coefficients under the null hypothesis. By the Central Limit theorem, one can show that the above Wald statistic has an asymptotic chi-square distribution with *r-s *degrees of freedom, under the null hypothesis. With finite sample sizes, our simulation results support the approximations by the stated chi-square distribution (not shown). Based upon this distribution, one can estimate the probability that quantifies the statistical significance in removing the *q*th SNP.

Two exceptional cases are worth mentioning. The first case is that if the *q*th SNP is in perfect LD with the remaining SNPs, removing that SNP would not alter the distribution of haplotypes. Consequently, estimated log ORs from the reduced haplotype analysis will be exactly the same as those in the full haplotype analysis, i.e., $\tilde{\beta }=\hat{\beta }$. Naturally, the above Wald-statistic equals zero, with zero degrees of freedom. In such a case, the SNP is removed without requiring a test. The second special case is that when only one SNP is in the model, the Wald statistic [2] degenerates to $\chi_{\text{1}}^{2}={\left(\hat{\beta }/\hat{\sigma }\right)}^{2}$.

#### A Forward Selection Procedure

**Figure 1:**
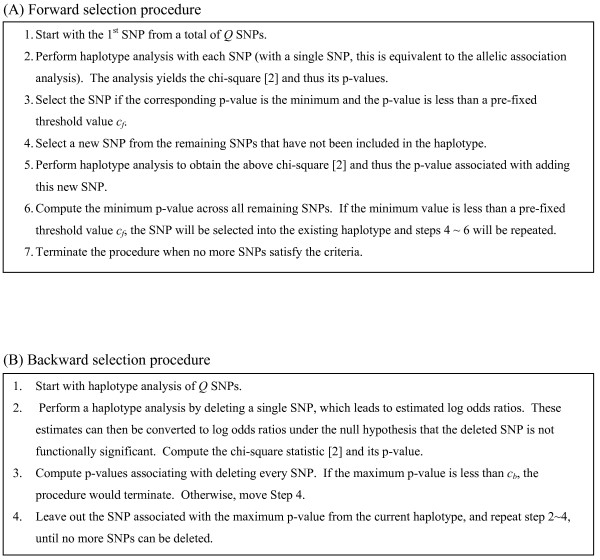
**Outline of the three computational algorithms for the stepwise selection of SNPs**. (A) Forward selection procedure. (B) Backward selection procedure. (C) Hybrid Selection Procedure.

Consider a haplotype analysis with *Q *SNPs from a gene or region. A forward selection procedure can be used to evaluate haplotypic associations with a single SNP, two SNPs, and progressively increasing numbers of SNPs. This procedure will be terminated when the minimum p-value exceeds a pre-set threshold (Figure 1A). Within this procedure, the threshold value *c*_*f *_is chosen to control false positive error rates. Here, we control the overall false positive error rate less than a pre-fixed rate, say 5% (further explored below).

#### A Backward Selection Procedure

While computationally efficient, the forward selection procedure may miss significant haplotypic associations due to variable haplotypic domains, i.e., the parameter domains are not necessarily hierarchical when a SNP is progressively reduced [6]. The desire to overcome this limitation motivates the backward selection procedure as an alternative to the forward selection procedure. The basic idea is to start with the haplotypes of all *Q *SNPs and then to eliminate irrelevant SNPs one SNP at a time (Figure 1B). The topic of choosing the threshold value *c*_*b *_for the backward selection is discussed further below.

#### A Hybrid Selection Procedure

While being generally preferred, the backward selection procedure can be time-consuming and prohibitive when the number of SNPs to be analyzed is large and each haplotype-based association analysis requires a substantial computation. To overcome this challenge, one may consider a hybrid selection procedure, i.e., combining both forward and backward selection procedures, patterning after the usual stepwise regression approach. One possible strategy is to initially use the forward selection procedure to add SNPs into haplotypes, and then to de-select those "selected SNPs" from established haplotypes with the backward selection procedure. The hybrid procedure, involving forward and backward selection threshold values *c*_*f *_and *c*_*b*_, stems directly from those described above (Figure 1C).

#### Permutation-Based Assessment of False Positive Error Rates

As noted above, the threshold values *c*_*f *_and *c*_*b *_for forward and backward selection procedures, respectively, are closely connected with false positive error rates, and stringent threshold values correspond to low false positive error rates. Choosing threshold values can be a challenge due to multiple comparisons with a series of highly correlated chi-square tests. The correlatedness among these tests can not be easily quantified due to varying levels of LD within a gene or within a specific region. However, a simple Bonferroni correction ignoring the correlation could lead to excessively conservative results.

We propose to use a permutation-based assessment to evaluate the false positive error rate (FPER), based on which the corresponding threshold value is chosen. Without requiring any distributional assumptions, the basic idea is to permute disease phenotypes across all subjects to create samples that could arise from the null hypothesis, in which SNPs have no associations with the disease phenotype. Thus, analysis of the permuted data, utilizing either the forward or backward procedure or a hybrid of both, would yield relevant statistics, in particular, p-values. Following analysis of the permuted data, the number of false positive errors is counted based on the pre-chosen threshold values (*c*_*f *_or *c*_*b*_). Repeating the permutation, say, 1000 times results in a sample of false-positive error counts. The average value over all permutations is taken as an estimate of the FPER. Thus, the threshold value (*c*_*f *_or *c*_*b*_) is chosen in such a way that the ultimate FPER is controlled at the pre-fixed rate.

### Simulation Studies

**Figure 2:**
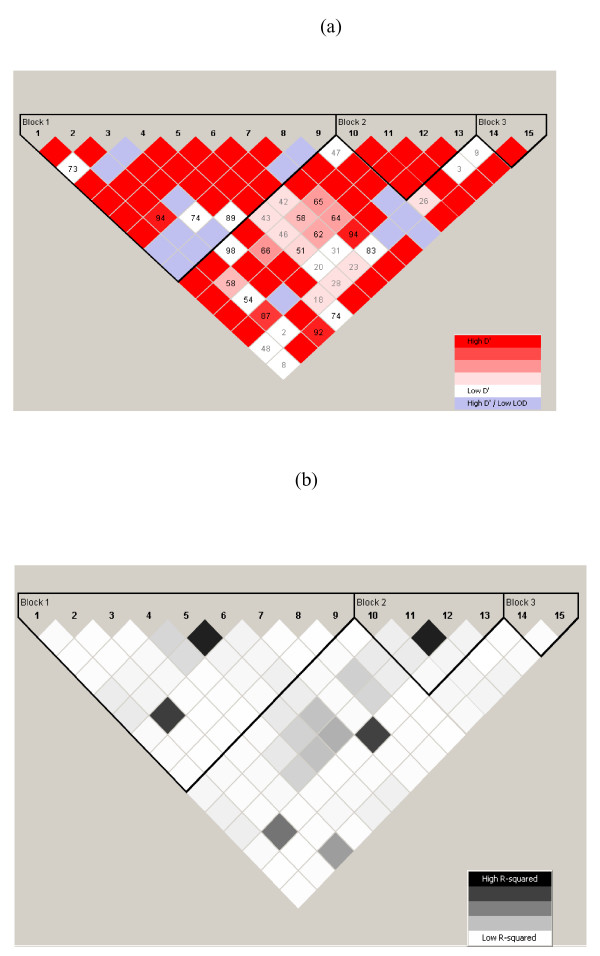
**LD patterns among 15 simulated SNPs and their haplotype block boundaries, indicated by solid lines**: (a) Standard D'/LOD pattern is shown, and (b) the LD pattern with r^2 ^using confidence intervals.

Simulation studies were conducted under the null hypothesis and also under alternative hypotheses. Assuming a typical coalescent process, we simulated 15 SNPs in varying degrees of LD, which were then used for the procedure (Figure 2). Under the null hypothesis, the simulated phenotype had no association with any simulated SNPs. For alternative hypotheses, we considered two different scenarios: 1) the phenotype was associated with a single SNP, and 2) the phenotype was associated with a haplotype of two SNPs.

Using a coalescent model, we generated genotype data under a typical evolutionary process, based upon Hudson's coalescent simulation program [15]. For the simulation, we specified a scaled recombination rate of 10, i.e., 4× (number of generations) × (recombination rate) to generate 2,000 haplotypes and with 150 segregation sites. From the 150 segregation sites, we selected 15 SNPs that had a minor allele frequency greater than 5%. Based upon 2,000 simulated haplotypes, we computed the haplotype frequencies of the 15 SNPs to represent the "true" haplotype frequencies in the study population. In this particular population, we observed 19 unique haplotypes with frequencies exceeding 0.1% (Table 1).

**Table 1:** Distribution of 19 simulated haplotypes

*Haplotype*	*Frequency*
000001001011000	0.2370
100001001011000	0.1440
000010001000000	0.1025
000010001111010	0.0900
000001001111000	0.0790
001001001011000	0.0565
010001101011100	0.0560
000001011000001	0.0525
000110001000000	0.0430
000000000000000	0.0305
000010001011100	0.0275
000110001010000	0.0275
000001101011100	0.0150
010001001011000	0.0095
000000001011000	0.0085
000001001000000	0.0085
000010001010000	0.0065
010001101000001	0.0050
000010001011010	0.0010

The following procedure was used to simulate the study population of one million people: we randomly drew a pair of haplotypes from the above haplotypic distribution to form individual diplotypes, but stripped away the phase information during this process. Using the penetrance function [1], with parameters specified corresponding to the null hypothesis and various alternative hypotheses, we computed the probability of an individual developing the disease. Based upon computed probabilities, we then simulated a binary disease status by the Bernoulli process. This simulation process was repeated one million times, resulting in the targeted study population. We also simulated two demographic variables: gender and age. For gender, we assumed that men and women were equally represented in the population. We assumed age to be uniformly distributed from 20 to 80 years. Under these assumptions, we randomly assigned gender and age to all individuals in the simulated study population

From the simulated study population, we randomly drew equal numbers of cases and controls to form case-control data sets. We considered different sample sizes in the simulation to test sample size effects. For each configuration of parameters, we repeated the simulations 1,000 times. To ensure the validity of the simulated results irrespective of SNP choices, we randomly selected one or two adjacent SNPs as functional SNPs in each replication. For each simulated data set, we used the HBSP to identify the functional SNP or haplotype and verified the finding. If the finding matched the functional element, it was a true positive finding. The percentages of true findings among the 1,000 replicates were recorded to quantify the true discovery rate (TDR), i.e., the percentage of true SNP associations identified. Since some SNPs were in LD with each other, we treated the discovery of SNP association, if it was at high LD with the true functional SNP (*D*^' ^≥ 0.8), as an acceptable discovery. To quantify this imperfect discovery, we introduced a statistic, the imperfect true discovery rate (iTDR). If the discovered SNP was neither the causal SNP nor at high LD with the causal SNP, it was counted as a false positive finding. Ideally, the rate of such false positive errors would be largely comparable to the FPER, which was controlled as described above.

## Results

### Simulated Data

#### Null Hypothesis

Under the null hypothesis, log ORs related to the haplotypes in the penetrance function [1] are set to 0. For gender and age, the coefficients were set at 0 and 0.01, respectively, with an intercept of 1. Sample sizes varied from 500 to 2000, with an equal number of cases and controls. Results of the simulation are reported in the first row of Table 2. The estimated FPER did not significantly deviate from 0.05, which was the chosen threshold, across the three sample sizes.

**Table 2:** False positive error rates are estimated under the null hypothesis.

Odds			Sample Sizes		
Ratios			250 × 2	500 × 2	1,000 × 2
Null Hypothesis					
			4.6	5.2	4.9
					
Alternative Hypothesis with a Single Functional SNP: 0 (ref) and 1					
1.10			1.0/1.0/5.8	13.6/13.6/6.4	23.2/27.9/8.3
1.20			13.7/17.2/7.3	31.0/37.9/5.5	47.2/56.3/6.7
1.30			19.0/23.8/4.9	46.7/51.6/9.0	62.5/75.0/7.3
1.40			42.5/44.7/8.0	68.5/74.0/6.5	75.7/82.0/6.8
1.50			57.6/64.4/6.5	77.8/80.0/7.0	83.0/88.0/6.1
2.00			77.0/84.0/6.7	90.0/90.8/4.9	93.8/93.8/5.6
3.00			90.5/92.9/4.8	94.8/95.5/6.1	96.4/97.0/5.2
5.00			92.1/93.5/4.3	96.3/96.3/4.9	97.5/98.0/5.0
Alternative Hypothesis with Two SNPs: 00 (ref), 01, 10 & 11					
Scenario 1					
1.0	1.3	1.0	17.6/41.2/7.3	42.9/67.8/6.0	39.4/71.1/9.0
1.0	1.5	1.0	30.9/57.1/9.1	44.4/76.1/8.0	45.9/72.7/10.0
1.0	2.0	1.0	57.5/90.0/5.3	62.2/95.0/4.0	79.9/99.3/1.3
1.0	3.0	1.0	61.2/96.3/4.7	63.0/98.0/2.0	89.0/99.0/1.0
1.0	5.0	1.0	64.8/97.8/3.3	66.7/99.7/1.4	100/100/2.0
Scenario 2					
1.0	1.0	1.3	20.0/46.3/5.0	26.1/60.0/6.1	30.7/69.2/7.5
1.0	1.0	1.5	16.7/50.0/4.0	23.5/52.9/6.0	29.2/78.4/6.5
1.0	1.0	2.0	10.0/55.6/5.5	20.0/66.7/6.5	36.0/80.1/6.3
1.0	1.0	3.0	20.5/74.7/8.0	25.7/74.6/6.4	34.4/81.0/7.0
1.0	1.0	5.0	31.6/80.8/9.3	32.9/82.1/6.2	33.3/85.2/6.0
Scenario 3					
1.0	1.3	1.3	20.8/54.1/7.3	21.6/59.5/10.1	40.4/67.4/8.1
1.0	1.3	1.5	22.9/47.6/7.3	38.3/78.5/6.0	39.5/79.8/8.0
1.0	1.3	2.0	15.6/48.5/7.6	32.0/79.0/6.3	44.5/85.3/7.3
1.0	1.3	3.0	26.7/77.4/6.3	38.7/80.1/6.2	50.7/91.3/4.6
1.0	1.3	5.0	30.3/78.7/6.6	41.3/82.6/6.0	55.5/91.0/4.4
Scenario 4					
1.0	1.5	1.3	44.4/72.2/6.7	45.9/79.7/10.0	53.3/88.7/7.1
1.0	1.5	1.5	50.0/75.0/6.6	45.7/80.6/9.2	47.8/87.3/7.0
1.0	1.5	2.0	33.9/69.5/9.7	37.5/79.4/8.3	49.7/93.7/6.3
1.0	1.5	3.0	28.1/74.6/9.0	42.1/82.0/6.7	51.0/94.9/6.0
1.0	1.5	5.0	39.4/86.2/7.7	49.2/94.89/5.7	53.0/99.4/5.0
Scenario 5					
2.0	1.5	1.3	50.1/82.1/5.3	51.9/85.8/7.0	56.3/90.1/7.5
2.0	1.5	1.5	51.7/89.4/7.3	52.4/90.0/7.0	57.4/93.5/6.7
2.0	1.5	2.0	52.6/89.3/9.3	51.0/92.8/6.3	60.0/99.7/5.0
2.0	1.5	3.0	52.7/89.3/9.0	54.1/90.3/6.2	65.4/100/4.8
2.0	1.5	5.0	53.8/89.6/5.3	55.3/96.7/5.3	70.1/100/4.7

Also estimated, under alternative hypotheses with either a single SNP or two SNPs, are the true discovery rate (TDR), imperfect true discovery rate (iTDR) and false positive error rate (FPER).

#### Alternative Hypothesis with a Single Functional SNP

Under the alternative hypothesis with a single but randomly chosen SNP, we specified related ORs as 1.10, 1.20, 1.30, 1.40, 1.50, 2.00, 3.00 and 5.00 in the penetrance function [1], to correspond to associations ranging from weak to strong. Table 2 lists the estimated TDR, imperfect true discovery rate iTDR, and FPER percentages for each value. As expected, the TDR increased with increasing sample size, 57.6% to 77.8% and then to 83.0% to detect OR = 1.5, as the sample size increased from 500 to 1000 and to 2000, respectively. Similarly, the TDR increased with the increasing OR values, 13.6% to 77.8% and then to 96.3% to detect ORs = 1.1, 1.5 and 5.0, respectively, with the sample size fixed at 1000. As expected, estimated FPER values were largely around 5%, with a few exceptions. Some minor elevations in FPER were probably due to weak LD with the functional SNP.

#### Alternative Hypothesis with Two Functional SNPs

Under the alternative hypothesis with two associated SNPs, we randomly chose two adjacent SNPs; they form four possible haplotypes (00, 01, 10, 11) with 00 as the reference haplotype. We created five different scenarios assuming different ORs for different haplotypes. Under the first scenario, the OR corresponding to haplotype 10 took values ranging from 1.3 to 5.0, which is similar to the single SNP situation. As expected, the TDR, iTDR and FPER estimates were comparable to those under the single SNP alternative hypotheses. Specifically, a case-control study of 500 subjects would have a 57.5% chance of detecting the true functional SNP with an OR of 2.0. At an increased sample size of 2000, the study would be able to detect an OR of 2.0 with nearly 80% TDR. The greater iTDR was mostly due to more than two SNPs being at high LD with these two functional SNPs. FPER values were generally less than 5%, with a few exceptions, partly because some SNPs had weak LD with the functional SNPs.

Under the second scenario, the OR corresponding to haplotype 11 varied from 1.3 to 5.0. While FPER estimates were around 5%, the TDR of detecting such associations was quite low, ranging from 10% to 36% for detecting OR of 2.0 with sample size varying from 500 to 2000, respectively. The primary reason for the reduced power was that the haplotype frequency for haplotype 11 was much lower than the others. Again, iTDR values were much greater than comparable TDR values, for the same reason stated above. When we increased the OR associated with haplotype 10 to 1.3 (scenario 3) and 1.5 (scenario 4), the TDR appreciably increased.

Under the fifth scenario, three OR values for three haplotypes deviated from 1.0, and both the TDR and the iTDR for detecting such genetic associations by the HBSP became very powerful. Even with a sample size of 500 subjects, the study had a 50~54% TDR and an 82~90% iTDR for detection of the true SNP-haplotype associations with the stated OR values.

### BPI Clinical Data

In our earlier analysis of a discovery cohort (N = 393), we identified BPI as an important candidate gene for the development of HCT-related AFO [14]. BPI was tagged by eight SNPs (C2738G, G7258A, G9083C, A10214G, G17016G, C23356T, A33065G, G36045A). Three haplotypes, tagged by these SNPs, were found to be significantly associated with the phenotype. Repeat analysis of these tagging SNPs in an independent validation cohort (N = 209) again revealed that multiple tagging SNP haplotypes were significantly associated with the AFO phenotype [14]. In the interest of reducing the number of SNP markers necessary to identify at-risk patients in clinical practice, we applied the proposed algorithm to find the most informative tagging SNPs. We applied the forward, backward and hybrid procedures to the discovery cohort, while adjusting for clinical covariates that were previously identified as important clinical risk factors for HCT-related AFO (age at transplant, pretransplant one-second forced expiratory volume, extent of graft versus host disease, and duration of follow-up). All three models identified the same two SNPs (C23356T and A33065G) as the most significantly associated with the disease phenotype (Table 3). With TG as the reference haplotype, the CA, CG, and TA haplotypes had ORs ranging from 1.52, 1.75 and 3.36, respectively (p-values 0.027, 0.014 and < 0.001, respectively). In the validation cohort, analysis of these SNPs again confirmed the statistical significance, with the exception of the TA haplotype. All ORs were comparable to those in the discovery cohort. These results confirmed that our approach can be applied to clinical data to identify the most informative SNP markers across a genetic region of significance.

**Table 3:** Estimated haplotype frequencies of two SNPs (C23356T and A33065G), estimated log odds ratios and their standard errors for all common haplotypes formed by identified SNPs

**Haplotype**	**Control Freq.**	**Case Freq.**	**Coef.**	**Odds Ratio**	**95% CI**	**Z-Score**	**P-value**
	Discovery Cohort (363 patients)						
TG	0.33	0.21		1.00	(reference)		
CA	0.39	0.42	0.42	1.52	(1.05, 2.21)	2.21	0.027
CG	0.21	0.23	0.56	1.75	(1.12, 2.72)	2.46	0.014
TA	0.07	0.15	1.21	3.36	(1.77, 6.37)	3.71	0.000
							
	Validation Cohort (209 patients)						
TG	0.34	0.20		1.00	(reference)		
CA	0.40	0.49	0.68	1.98	(1.19, 3.27)	2.65	0.008
CG	0.18	0.23	0.75	2.11	(0.99, 4.50)	1.94	0.053
TA	0.09	0.09	0.63	1.87	(0.67, 5.23)	1.20	0.230

## Discussion

A complementary approach to the HBSP is the direct application of the stepwise regression approach to assess disease associations with SNP alleles or genotypes at multiple SNP loci, as described by Clayton and his colleagues [3,16]. Basically, this approach treats individual SNP alleles or genotypes as covariates and then assesses their associations with the disease phenotype via the logistic regression model [1]. To identify functional SNPs, they propose using the usual stepwise regression technique to systematically analyze all SNP genotypes and their cross products. Those cross-product terms, if significant, are surrogates for possible haplotypic associations. While its key advantage includes the simplicity and familiarity to most population researchers, the interpretation of cross-product terms as possible haplotypic associations is not straightforward. Moreover, such an approach does not take full advantage of extended common haplotypes across many SNPs, because one has to numerate all cross products to detect a high-order interaction; e.g., eight SNPs will create 256 (= 2^8^) allelic cross products or 6,561 (= 3^8^) genotypic cross products.

To compare both stepwise approaches, we utilized simulation studies to assess the TDR, iTDR and FPER for the stepwise regression approach. We conducted the simulation studies under both null and alternative hypothesis and used the same 15 simulated SNPs from the simulation study population generated for our proposed approach. The simulation results are reported in Table 4. Under scenario 1, the OR corresponding to haplotype 10 takes values ranging from 1.3 to 5.0, and the FPER and iTDR estimates from the usual approach were comparable to those from the HBSP. However, the TDRs for detecting associations with the usual stepwise regression technique were lower than those obtained with HBSP (Table 2). Under scenario 2, the OR corresponding to haplotype 11 varies from 1.3 to 5.0. While the FPER estimates were around 5%, the TDR for detecting associations was quite low, even though the sample size reached 2000. Thus, compared to HBSP, the usual stepwise regression technique has less power for detecting true genetic associations and compatible power in discovering imperfect true genetic associations.

**Table 4:** False positive error rate under the null hypothesis, and true discovery rate (false positive error rate) under alternative hypotheses 1 when the Clayton's stepwise approach is used to select a subset of SNPs.

Odds			Sample Sizes		
Ratios			250 × 2	500 × 2	1,000 × 2
Null Hypothesis					
			5.5	5.2	5.0
Alternative Hypothesis with a Single Functional SNP: 0(ref) and 1					
1.5			65.7/70.0/5.5	71.3/87.0/5.1	74.3/100/5.0
2.0			71.3/100/5.3	71.1/100/5.0	85.0/100/5.0
3.0			80.7/100/5.0	89.3/100/5.0	94.0/100/5.0
Alternative Hypothesis with Two SNPs: 00 (ref), 01, 10 & 11					
Scenario 1					
1.0	1.3	1.0	14.3/51.0/6.1	31.3/68.8/5.7	32.1/77.0/6.3
1.0	1.5	1.0	29.2/61.0/7.0	42.2/72.1/7.1	33.2/78.2/7.2
1.0	2.0	1.0	36.5/92.2/6.0	51.7/95.0/5.3	71.2/100/4.4
1.0	3.0	1.0	51.1/98.2/5.0	60.3/98.8/4.1	82.4/99.8/4.0
1.0	5.0	1.0	52.0/99.1/4.3	61.2/100/4.0	100/100/4.0
Scenario 2					
1.0	1.0	1.3	7.9/45.2/5.5	21.4/65.0/5.4	19.7/71.2/6.3
1.0	1.0	1.5	7.8/51.4/5.3	14.5/55.1/5.2	21.7/80.1/6.3
1.0	1.0	2.0	7.2/55.2/6.1	19.9/74.5/6.0	30.0/82.1/6.0
1.0	1.0	3.0	17.8/93.0/6.0	23.4/94.4/5.4	25.4/95.7.0/5.4
1.0	1.0	5.0	23.4/90.8/5.7	32.9/88.1/6.2	34.3/89.2/5.4

For illustrative purposes, we have also applied stepwise logistic regression models to the BPI discovery cohort. The forward stepwise selection procedures did not detect any SNPs at the significance level of *α *= 0.05. The backward stepwise elimination procedure detected the joint effect of the two SNPs (C23365T and A33065G), the results from which were consistent to those obtained by HBSP.

Recently, Cheng and colleagues have described another method of mapping functional sites with SNP-haplotypes [17], which shares a similar scientific objective to the HBSP. The key idea underlying their approach is to compute an overall statistic that summarizes all associations within a sliding window of multiple SNPs. Systematically covering genes/regions with sliding and overlapping windows, their method can pinpoint one or more sites that are functionally associated with the disease phenotype. The end result of this approach is to identify functional sites, which is complementary to that of the HBSP. In fact, one can apply the HBSP to produce the overall statistic for each sliding window, as a way of detecting haplotypic associations in an efficient manner.

While the HBSP performed well for the studies reported in this paper, its function can also be extended to other types of analysis. The HBSP can be adapted for other phenotypes, such as continuous, censored or categorical, via their corresponding link functions. As presented above, the key statistic needed to construct the Wald statistic [2] is the covariance matrix of all estimated parameters, which is typically obtainable from the maximum likelihood or estimating equations. In addition, the current method is structured to detect major genetic associations via the assumed penetrance model [1], and is not designed to detect gene-environmental or gene-gene interactions. To achieve those objectives, we can extend the penetrance model by including those interactions, which have been elaborated elsewhere [4].

## Conclusion

The HBSP described above is effective in selecting a subset of SNPs whose haplotypes are significantly associated with a disease phenotype by eliminating SNPs with random polymorphisms. The HBSP retains the advantages of haplotype-based analysis while minimizing the deficiencies associated with typical haplotype-based analysis that includes extraneous SNPs. Simulation studies indicated that our permutation scheme effectively controls the false positive error rate while HBSP has adequate power to identify those functional SNPs/haplotypes. The illustrative example with SNP data from the BPI gene in a transplant cohort demonstrated the success of the HBSP in identifying two consecutive SNPs out of eight SNPs from the discovery cohort and in validating their associations with the disease phenotype from clinical data.

## Authors' contributions

YY carried out numerical calculations and simulations, SL implemented the methodology and simulation strategy, JC contributed BPI data and helped to interpret results, JA participated in the manuscript preparation, LZ conceived the idea, led the development and simulation, and participated in the manuscript preparation. All authors read and approved the final manuscript.
